# Supplementary material for: Identification of photoperiod-induced specific miRNAs in the adrenal glands of Sunite sheep (Ovis aries)
Source: Front Vet Sci. 2022 Jul 22;9:888207. doi: 10.3389/fvets.2022.888207 (PMC9354845; doi:10.3389/fvets.2022.888207)
Supplement: Supplementary file 1 [file Data_Sheet_1.ZIP › Supplementary file/miRDeep2 file.zip!/miRDeep2 file/Novel_Structure/SP42A3/Novel_579.pdf]

Provisional ID	:	NC_019477.2_26208
Score total	:	0.9
Score for star read(s)	:	-1.3
Score for read counts	:	-1.3
Score for mfe	:	2.1
Score for randfold	:	1.6
Score for cons. seed	:	
Total read count	:	9
Mature read count	:	9
Loop read count	:	0
Star read count	:	0

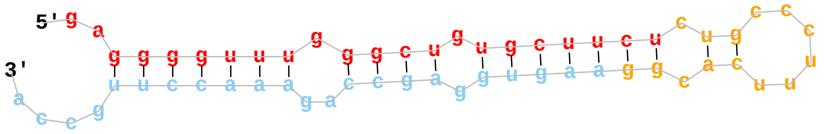

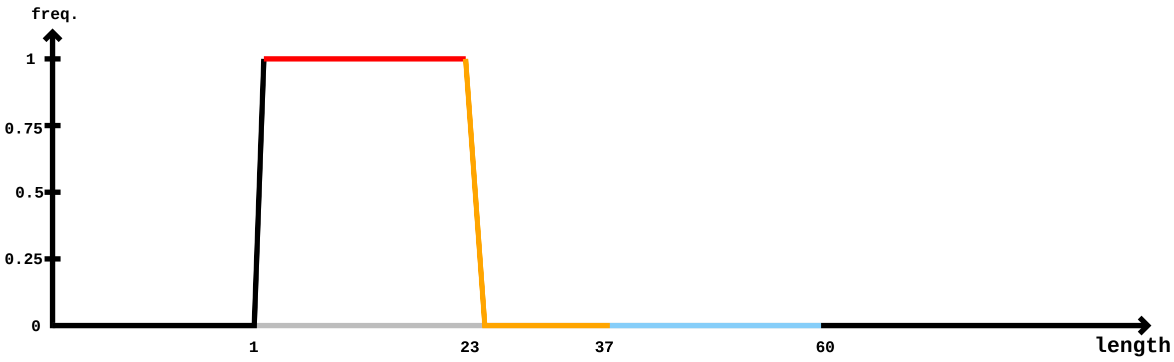


```
                    Mature                Star
5'- gaccugggcugcgucccauggagggguuugggcugugcuucucugcccuuucacggaaguggagccagaaaccuugccaaggccguggcugcugucuuucaugcagcaaggu   -3'    exp
    .((((.(((((((.((..(((.(((((((.((((.(((((((.((......)).))))))).))))..))))))).))).)).)))))))(((((.......))))).))))    reads    mm    sample
    ....................gagggguuugggcugugcuucu......................................................................    9        0     seq
```
